# The Microbiological Etiology of Fracture-Related Infection

**DOI:** 10.3389/fcimb.2022.934485

**Published:** 2022-07-07

**Authors:** Melissa Depypere, Jonathan Sliepen, Jolien Onsea, Yves Debaveye, Geertje A. M. Govaert, Frank F. A. IJpma, Werner Zimmerli, Willem-Jan Metsemakers

**Affiliations:** ^1^ Department of laboratory medicine, University Hospitals Leuven, Leuven, Belgium; ^2^ Department of Microbiology, Immunology and Transplantation, Laboratory of Clinical Bacteriology and Mycology, KU Leuven, Leuven, Belgium; ^3^ Department of Trauma Surgery, University Medical Center Groningen, Groningen, Netherlands; ^4^ Department of Trauma Surgery, University Hospitals Leuven, Leuven, Belgium; ^5^ Department of Development and Regeneration, KU Leuven - University of Leuven, Leuven, Belgium; ^6^ Department of Intensive Care Medicine, University Hospitals Leuven, Leuven, Belgium; ^7^ Department of Trauma Surgery, University of Utrecht, University Medical Center Utrecht, Utrecht, Netherlands; ^8^ Basel University Medical Clinic, Kantonsspital Baselland, Liestal, Switzerland

**Keywords:** fracture, infection, fracture-related infection, microbiology, antibiotic resistance

## Abstract

**Purpose:**

Fracture-related infection (FRI) is an important complication related to orthopaedic trauma. Although the scientific interest with respect to the diagnosis and treatment of FRI is increasing, data on the microbiological epidemiology remains limited. Therefore, the primary aim of this study was to evaluate the microbiological epidemiology related to FRI, including the association with clinical symptoms and antimicrobial susceptibility data. The secondary aim was to analyze whether there was a relationship between the time to onset of infection and the microbiological etiology of FRI.

**Methods:**

FRI patients treated at the University Hospitals of Leuven, Belgium, between January 1st 2015 and November 24th 2019 were evaluated retrospectively. The microbiological etiology and antimicrobial susceptibility data were analyzed. Patients were classified as having an early (<2 weeks after implantation), delayed (2-10 weeks) or late-onset (> 10 weeks) FRI.

**Results:**

One hundred ninety-one patients with 194 FRIs, most frequently involving the tibia (23.7%) and femur (18.6%), were included. *Staphylococcus aureus* was the most frequently isolated pathogen, regardless of time to onset (n=61; 31.4%), followed by *S. epidermidis* (n=50; 25.8%) and non-*epidermidis* coagulase-negative staphylococci (n=35; 18.0%). Polymicrobial infections (n=49; 25.3%), mainly involving Gram negative bacilli (GNB) (n=32; 65.3%), were less common than monomicrobial infections (n=138; 71.1%). Virulent pathogens in monomicrobial FRIs were more likely to cause pus or purulent discharge (n=45;54.9%; p=0.002) and fistulas (n=21;25.6%; p=0.030). Susceptibility to piperacillin/tazobactam for GNB was 75.9%. Vancomycin covered 100% of Gram positive cocci.

**Conclusion:**

This study revealed that in early FRIs, polymicrobial infections and infections including Enterobacterales and enterococcal species were more frequent. A time-based FRI classification is not meaningful to estimate the microbiological epidemiology and cannot be used to guide empiric antibiotic therapy. Large multicenter prospective studies are necessary to gain more insight into the added value of (broad) empirical antibiotic therapy.

## Introduction

Fracture-related infection (FRI) is a serious complication following skeletal injury (Depypere et al., 2019b; Metsemakers et al., 2019). Although consensus guidelines regarding the diagnosis and treatment of FRI were published (Depypere et al., 2019a; Depypere et al., 2019b; Foster et al., 2020; Govaert et al., 2020), knowledge gaps remain. An important example is the microbiological epidemiology of FRI where, as opposed to periprosthetic joint infection (PJI) (Tsukayama et al., 1996; Carrega et al., 2008; Sharma et al., 2008; Benito et al., 2016; Drago et al., 2017; Triffault-Fillit et al., 2019), data are limited. Although PJI and FRI are both implant-related infections, there are some important differences between these entities that could influence the type of microbiological flora that is present at time of diagnosis. A first difference is the initial damage to the soft tissues overlying the surgical site. An open fracture potentially leads to wound contamination with soil microorganisms, and massive crush injuries may cause disturbed vascularization with concomitant skin necrosis. For these reasons, a wider range of pathogens is expected as compared to PJI, where the device is implanted in a sterile environment. A second difference is the presence of a fracture and the need for biomechanical stability. While stability is important for both the prevention and treatment of FRI, it is not clear whether it influences the type of local infecting agents (Foster et al., 2021). Although data on the topic is limited, a recent study showed that there is no significant difference in pathogen distribution between FRI and PJI (Rupp et al., 2021).

The Willenegger and Roth classification represents the time-dependent pathophysiologic changes of FRI (Willenegger and Roth, 1986; Metsemakers et al., 2019). This classification is based on time after device implantation. It classifies FRIs in early (< 2 weeks), delayed (2-10 weeks) and late-onset infections (> 10 weeks). However, evidence for such a clear, time-based cut-off to aid in the decision-making process is scarce. A recent study showed that time to onset of FRI is not the only treatment-guiding factor in the decision-making process towards the choice of surgical strategy (Morgenstern et al., 2021). Little is known about the value of this classification regarding the microbiological spectrum at time of debridement. A recent publication did not report significant differences in the pathogen distribution between the three subgroups (Baertl et al., 2022).

We performed a study based on two aims. The primary aim was to evaluate the microbiological epidemiology of FRIs at our center, including the association with clinical symptoms and antimicrobial susceptibility data. The secondary aim was to analyze whether there was a relationship between the Willenegger and Roth classification and the microbiological etiology of FRI which could guide empiric antibiotic therapy.

## Patients and Methods

### Study Design and Inclusion/Exclusion Criteria

This retrospective cohort study evaluated data of patients with an FRI who were treated between January 1^st^ 2015 and November 24^th^ 2019 at the Department of Trauma Surgery of the University Hospitals Leuven (Belgium). All patients were treated according to recommendations of the multidisciplinary team. The multidisciplinary team consisted of trauma- and plastic surgeons, microbiologists, clinical pharmacists, radiologists/nuclear medicine physicians and clinical infectious disease specialists. All consecutive patients were identified from the operating theater logbooks, and all case notes were retrieved. Patient data was collected using the hospital electronic patient file system and included in the study database. The diagnosis of FRI was determined according to the criteria of the FRI consensus definition (Metsemakers et al., 2018). Exclusion criteria were patients with an FRI diagnosed outside the study period, patients younger than 18 years of age, pathological fractures, fractures of the skull and fractures of the spine. To reduce data misinterpretation and data entry mistakes, the retrospective review of medical records was carried out by two of the authors (JS, JO). All patient charts were searched for a complete medical and microbiological history. The latter was verified by two other authors (MD, WJM).

### Ethical Statement

The study protocol was conducted following good clinical practice guidelines. The study was approved by the Ethics Committee of the University Hospitals Leuven, Belgium (S62394).

### Microbiological Analysis

When patients were suspected of having an FRI, at least five tissue biopsies were taken during a surgical procedure and incubated in Wilkins-Chalgren broth for seven days. Every day, broths were checked for cloudiness. When cloudy, Gram-staining was performed, and suitable agars were streaked and incubated. In cases without cloudiness, broths were streaked on chocolate agar and anaerobic blood agar plates. Identification was performed using Matrix-assisted laser desorption ionization Time-Of-Flight mass spectrometry (Maldi-TOF MS) (Bruker, Bremen, Germany). Antibiotic susceptibility was tested on Vitek2 (BioMérieux, Marcy l’Etoile, France), and interpreted according to European Committee on Antimicrobial Susceptibility Testing (EUCAST) breakpoints since August 2017. Before this date, Clinical and Laboratory Standards Institute (CLSI) breakpoints were applied.

Two or more positive cultures with identical pathogens were considered confirmatory for infection (Metsemakers et al., 2018). Single positive culture tests were considered only when a virulent pathogen was isolated. Virulent pathogens were defined *a priori* as Gram negative bacilli (GNB), *Staphylococcus aureus, Staphylococcus lugdunensis*, enterococci, beta-hemolytic streptococci, *Streptococcus anginosus* group (previously milleri group streptococci), *Streptococcus pneumoniae* and *Candida* spp (Onsea et al., 2022). Single positive cultures with non-virulent pathogens were not further evaluated as they were seen as contaminants.

### Statistical Analysis

Data was collected and analyzed using SPSS (version 23, IBM Inc, Armonk, NY, USA). The data were reported using standard descriptive statistics, including counts and percentages to report proportions, mean and standard deviation (SD) for normally distributed continuous variables and median and Inter-Quartile Range (p_25_-p_75_) for non-parametric variables. Normality of continuous data was tested with the Shapiro-Wilk test and homogeneity of variances was tested using the Levene’s test. In case of parametric data, a one-way Analysis of Variance (ANOVA) or Student’s t-test (with either equal variances assumed or not) was used to compare differences between groups (based on time to onset of FRI). In case of non-parametric data, the Kruskal-Wallis or Mann-Whitney U test was used as appropriate. For non-continuous data Chi-square tests or Fisher exact tests were used as appropriate. P-values below 0.05 were considered statistically significant.

Data included for statistical analysis were age, sex, body mass index (BMI) and American Society of Anesthesiologists (ASA) score at time of clinical presentation. Furthermore, the anatomical site, Gustilo-Anderson type, confirmatory and suggestive diagnostic criteria and microbiological analysis were taken into account. The Gustilo-Anderson classification can be used to classify open fractures according to their severity. As mentioned earlier, FRIs were classified according to the Willenegger and Roth classification (Willenegger and Roth, 1986; Metsemakers et al., 2018).

## Results

### Population Characteristics

A total of 191 patients with 194 FRIs were included in this study. There were 65 (34.0%) women and 126 (66.0%) men, with a median age of 54 (p_25_-p_75_: 43-67) years. Most patients had an ASA score of 2 (n=97, 50.8%). The tibia (n=46, 23.7%) was the anatomical site most frequently involved, followed by the femur (n=36, 18.6%) and the ankle (n=30, 15.5%). Overall, 48 (24.7%) infections were related to an open fracture. **Table 1** shows the population characteristics and clinical presentation according to time to onset of FRI.

**Table 1:** Population characteristics and clinical presentation according to time after device implantation.

Characteristics	Early FRI (<14 days) n = 34 (%)	Delayed FRI (14-70 days) n = 74 (%)	Late FRI (>70 days) n = 86 (%)	p-value (early vs delayed vs late)
**Sex†**				0.534
Male	19 (57.6)	50 (67.6)	57 (68.6)	
Female	14 (42.4)	24 (32.4)	27 (32.4)	
**Age** median (p_25_-p_75_)	61 (44–70)	52 (41-70)	55 (43-67)	0.725
**BMI** median (p_25_-p_75_)	25.7 (23.2-30.3)	25.4 (23.8-28.4)	26.4 (22.7-30.2)	0.637
**ASA score^†^**				0.726
I	4 (12.1)	14 (18.9)	13 (15.5)	
II	18 (54.5)	34 (45.9)	45 (53.6)	
III	9 (27.2)	24 (32.4)	25 (29.8)	
IV	2 (6.0)	2 (2.7)	1 (1.2)	
**Fracture characteristics**				
**Localization**				
Clavicle	0 (0.0)	10 (13.5)*	3 (3.5)	***0.013***
Humerus	5 (14.7)	11 (14.9)	8 (9.3)	0.511
Forearm	0 (0.0)	6 (8.1)	10 (11.6)	0.094
Femur	7 (20.6)	6 (8.1)*	23 (26.7)*	***0.010***
Tibia	12 (35.3)	15 (20.3)	19 (22.1)	0.209
Fibula	3 (8.8)	9 (12.2)	6 (7.0)	0.527
Ankle	4 (11.8)	12 (16.2)	14 (16.3)	0.806
Calcaneus	0 (0.0)	2 (2.7)	2 (2.3)	1.000
Patella	2 (5.9)	2 (2.7)	1 (1.2)	0.262
Scapula	1 (2.9)	0 (0.0)	0 (0.0)	0.175
Sternum	0 (0.0)	1 (1.4)	0 (0.0)	0.557
**Open/closed**				0.067
Closed	21 (61.8)	61 (82.4)	64 (74.4)	
Open	13 (38.2)	13 (17.6)	22 (25.6)	
**Gustilo-Anderson type**				0.682
1	4 (11.8)	3 (4.1)	7 (8.1)	
2	4 (11.8)	6 (8.1)	11 (12.8)	
3	5 (14.7)	4 (5.4)	4 (4.7)	
**Clinical presentation**				
Fistula	7 (20.6)	16 (21.6)	19 (22.1)	0.984
Wound breakdown	8 (23.5)	27 (36.5)	17 (19.8)	0.053
Purulent discharge/pus	18 (52.9)	35 (47.3)	30 (34.9)	0.120
Redness	19 (55.9)	38 (51.4)	36 (41.9)	0.290
Pain	9 (26.5)	25 (33.8)	37 (43.0)	0.098
Swelling	16 (47.1)	23 (32.1)	31 (36.0)	0.275
Fever (≥38.3°C)	2 (5.9)	11 (14.9)*	2 (2.3)*	***0.008***
Local warmth	7 (20.6)	10 (13.5)	6 (7.0)	0.094
Joint effusion	3 (8.8)	7 (9.5)	9 (10.5)	0.956
Wound drainage	16 (47.1)	27 (36.5)	20 (23.3)*	**0.028**
**Microbiological characteristics**				
Monomicrobial	17 (50.0)*	58 (78.4)	63 (73.3)	**0*.009***
Polymicrobial	16 (47.1)*	14 (18.9)	19 (22.1)	***0.005***
Culture-negative	1 (2.9)	2 (2.7)	4 (4.7)	0.877
Time to onset median (p_25_-p_75_)	9 (6.75-11.25)	30 (18.75-42.0)	308 (148-607.25)	–

^†^Adds up to 191. Three patients had a second episode of FRI at a different anatomical location *Post-hoc testing showed statistically significant difference from the other groups at p < 0.05. p_25_-p_75_: 25^th^ and 75^th^ percentile, inter-quartile range.

### Microbiological Etiology

Microbiological cultures were performed in all 194 infectious cases. In seven patients, culture results were negative. Five of them were treated with antibiotics during the two weeks before sampling.

**Figure 1:**
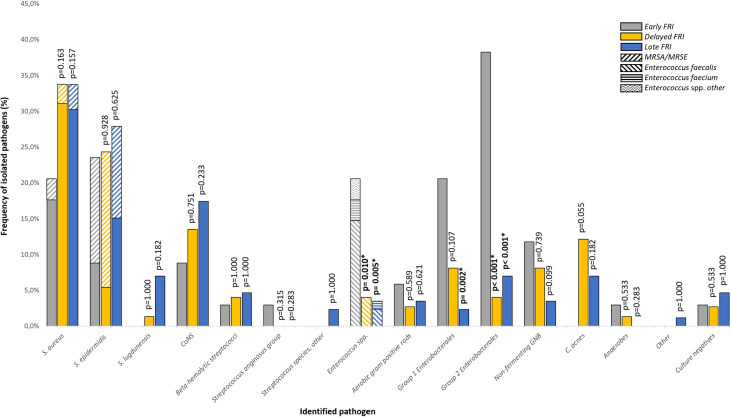
Microbiological etiology of FRI according to time to onset of FRI.

In open fractures, 13 (27.1%) GNB were isolated as compared to 28 (19.2%) in closed fractures. **Figure 1** shows the microbiological results according to time to onset of FRI. *S. aureus* was the most commonly isolated pathogen regardless of time to onset (n=61; 31.4%), followed by *S. epidermidis* (n=50; 25.8%) and non-*epidermidis/non-lugdunensis* coagulase-negative staphylococci (CoNS) (n=29; 14.9%). Methicillin-resistant *S. aureus* (MRSA) was isolated in six FRIs (3.1%). *Enterococcus* spp. was significantly more prevalent in the early FRI (n=7; 20.6%), as compared to the delayed (n=3; 4.1%; p=0.010) and late-onset group (n=3; 3.5%; p=0.005). Overall, *E. faecalis* was the most represented *Enterococcus* spp. (n=10; 5.2%). *Enterobacterales* were divided in group 1 and 2, with group 2 being pathogens which are intrinsically resistant to amoxicillin-clavulanic acid. In the early infection group, group 2 *Enterobacterales* were more prevalent (n=13; 38.2%) than in the delayed (n=3; 4.1%; p<0.001) and late infection group (n=6;7.0%, p<0.001), with *Enterobacter cloacae* as the most common pathogen. Group 1 *Enterobacterales* were found more frequently in the early infection group (n=7; 20.6%), as compared to the infections in the late-onset group (n=2; 2.3% p=0.002). Non-fermenting GNB were observed in thirteen cases (6.7%), of which *Pseudomonas aeruginosa* was the most prevalent pathogen (n=6; 46.2%). Although not statistically significant (p=0.055), the prevalence of *Cutibacterium acnes* tended to be higher in the delayed group (n=9; 12.2%) compared to the early onset group (n=0; 0%). Other anaerobes, *Streptococcus* spp. and aerobic Gram positive bacilli (GPB) were rarely detected.

**Figure 2:**
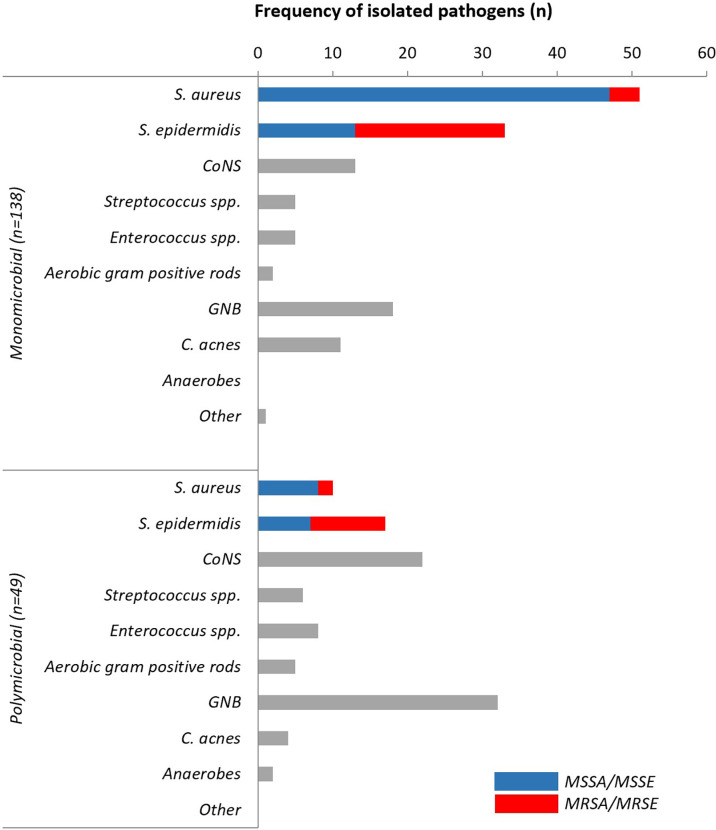
Microbiological epidemiology in mono- and polymicrobial FRIs. CoNS, Coagulase-Negative Staphylococci; GNB, Gram negative bacilli (Enterobacterales and non-fermenting GNB); C. acnes, *Cutibacterium acnes*; MSSA, methicillin-sensitive *Staphylococcus aureus*; MSSE, methicillin-sensitive *Staphylococcus epidermidis*; MRSA, methicillin-resistant *Staphylococcus aureus*; MRSE, methicillin-resistant *Staphylococcus aureus*.

Polymicrobial infections were diagnosed in 49 cases (25.3%) and mostly observed in the early onset group (n=16; 47.1%; p=0.005). The combinations of causative pathogens in polymicrobial infections are displayed in **Table 2**. In contrast, monomicrobial infections were more common overall (n=138; 71.1%), but statistically less frequent in the early onset group (n=17; 50.0%; p=0.009). The main microorganisms present in polymicrobial infections were GNB (n=32; 65.3%), followed by non-*epidermidis* CoNS (n=22; 44.9%) and *S. epidermidis* (n=17; 34.7%). In monomicrobial infections, the proportion of *S. aureus* was greater (n=51; 37.0%) than in polymicrobial infections (n=10; 20.4%; p=0.034). The second most common pathogen was *S. epidermidis* (n=33; 23.9%), followed by GNB (n=18; 13.0%) and non-*epidermidis* CoNS (n=13; 9.4%). **Figure 2** compares the microbiological epidemiology in mono- and polymicrobial FRI.

**Table 2:** The microbiological etiology of polymicrobial fracture-related infections.

No.	Fracture type	Pathogen 1	Pathogen 2	Pathogen 3	Pathogen 4	Pathogen 5
**1**	Closed	*Enterococcus faecalis*	*Enterobacter cloacae*			
**2**	Closed	*S. lugdunensis*	*C. acnes*			
**3**	Closed	Enterobacter cloacae*	Enterobacter cloacae*			
**4**	Closed	*S. epidermidis*	*S. capitis*	*Corynebacterium simulans*		
**5**	Closed	*S. epidermidis*	*Strep. agalactiae*	*Enterobacter cloacae*		
**6**	Closed	*S. epidermidis*	*Bacillus cereus*	*C. acnes*		
**7**	Closed	*Klebsiella pneumoniae*	*Enterococcus faecalis*	*Proteus mirabilis*		
**8**	Closed	*S. epidermidis*	*Enterobacter cloacae*	*S. pettenkoferi*		
**9**	Closed	*S. aureus*	*Enterobacter cloacae*			
**10**	Closed	*S. epidermidis*	*S. warneri*			
**11**	Closed	*S. aureus*	*S. capitis*			
**12**	Closed	*S. epidermidis*	*S. haemolyticus*			
**13**	Closed	*S. simulans*	*Enterobacter cloacae*			
**14**	Closed	*Proteus mirabilis*	*Enterobacter aerogenes*			
**15**	Closed	*Escherichia coli*	*Enterobacter cloacae*			
**16**	Closed	*S. saccharolyticus*	*S. lugdunensis*			
**17**	Closed	*Klebsiella pneumoniae*	*Proteus mirabilis*	*Strep. agalactiae*	*Strep. anginosus*	
**18**	Closed	*S. aureus*	*S. lugdunensis*			
**19**	Closed	*Peptoniphilus harei*	*S. capitis*			
**20**	Closed	*S. epidermidis*	*S. lugdunensis*	*S. capitis*		
**21**	Closed	*Enterobacter cloacae*	*Alcaligenes faecalis*			
**22**	Closed	*S. aureus*	*Strep. agalactiae*			
**23**	Closed	*S. epidermidis*	*Corynebacterium tuberculostearicum*			
**24**	Closed	*Citrobacter freundii*	*Klebsiella oxytoca*			
**25**	Closed	*S. epidermidis*	*S. capitis*			
**26**	Closed	*S. simulans*	*Acinetobacter* spp.			
**27**	Closed	*S. aureus*	*Pseudomonas aeruginosa*			
**28**	Closed	*Citrobacter koseri*	*Proteus mirabilis*			
**29**	Closed	*S. aureus*	*S. epidermidis*			
**30**	Closed	S. aureus*	S. aureus*			
**31**	Closed	*S. aureus*	*S. epidermidis*			
**32**	Closed	*S. aureus*	*Enterococcus faecalis*			
**33**	Closed	*S. capitis*	*C. acnes*			
**34**	Closed	*S. epidermidis*	*S. warneri*			
**35**	Closed	*S. auricularis*	*S. lugdunensis*	*S. capitis*	*C. acnes*	
**36**	Closed	*S. capitis*	*S. hominis*			
**37**	Open	*S. epidermidis*	*Enterococcus faecalis*			
**38**	Open	*S. epidermidis*	*Enterococcus faecium*	*S. pettenkoferi*		
**39**	Open	*Enterococcus mundtii*	*Pseudomonas putida*	*Enterococcus hirae*	*Clostridium* spp.	*Serratia fonticola*
**40**	Open	*Escherichia coli*	*Stenotrophomonas maltophilia*			
**41**	Open	*Escherichia coli*	*Enterococcus faecalis*	*Morganella morganii*	*Proteus mirabilis*	
**42**	Open	*Enterococcus faecalis*	*Enterobacter cloacae*			
**43**	Open	*Pseudomonas aeruginosa*	*Enterobacter cloacae*			
**44**	Open	*S. epidermidis*	*Bacillus cereus*			
**45**	Open	*Escherichia coli*	*Aeromonas hydrophilia*			
**46**	Open	*S. aureus*	*Strep. group C*	*Strep. agalactiae*		
**47**	Open	*S. schleiferi*	*Acinetobacter* spp.			
**48**	Open	*Strep. mitis*	*Enterobacter cloacae*			
**49**	Open	*S. aureus*	*Pseudomonas aeruginosa*			

*Different strains of same pathogen; S., Staphylococcus; Strep., Streptococcus; C., Cutibacterium.

**Figure 3:**
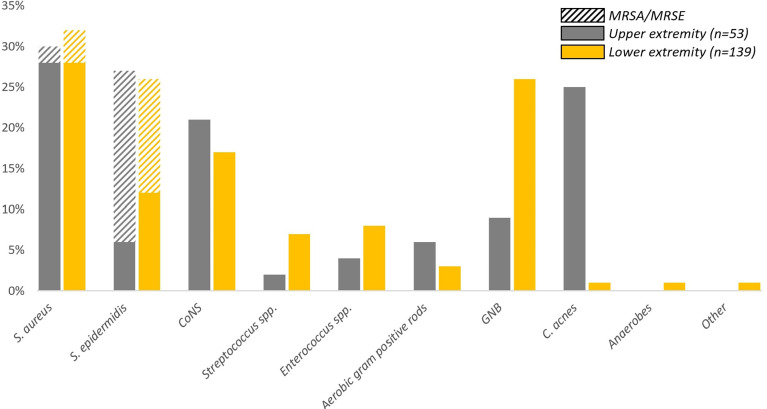
Frequency of pathogens isolated per body region. Upper extremity: humerus and forearm; lower extremity: femur, tibia, fibula, patella, ankle and foot. Only two patients suffered an FRI of the axial skeleton, these patients were excluded from visualization in this figure as the percentages would be misleading. The cultured pathogen in the axial FRI group was a *S. aureus* in one patient and a *S. epidermidis* in the other.

**Figure 3** shows the distribution of pathogens according to body region. *C. acnes* is predominantly isolated from the upper extremity (13/53; 24.5%) compared to the lower extremity (2/139; 1.4%) and the axial skeleton (0/2; 0%). There is no notable difference between the presence of *S. aureus*, *S. epidermidis* and other CoNS when comparing lower and upper extremity FRIs.

### Clinical Presentation

The association between highly virulent pathogens and clinical confirmatory signs in monomicrobial infections is shown in **Table 3**. A statistically significant association was found for the combination of pus/purulent drainage (n=45; 54.9%, p=0.002) and fistula (n=21; 25.6%; p=0.030), with the presence of highly virulent pathogens. For wound breakdown there was no significant association.

**Table 3:** The association between virulent pathogens in monomicrobial infections and clinical confirmatory signs.

	Virulent pathogen		P-value
	Yes n (%)	No n (%)	
**Pus/purulent discharge**	45 (54.9)	16 (28.6)	0.002*
**Fistula**	21 (25.6)	6 (10.7)	0.030*
**Wound breakdown**	23 (28.0)	9 (16.1)	0.102

*Statistically significant at p<0.05.

*Antimicrobial susceptibility data*

**Figure 4:**
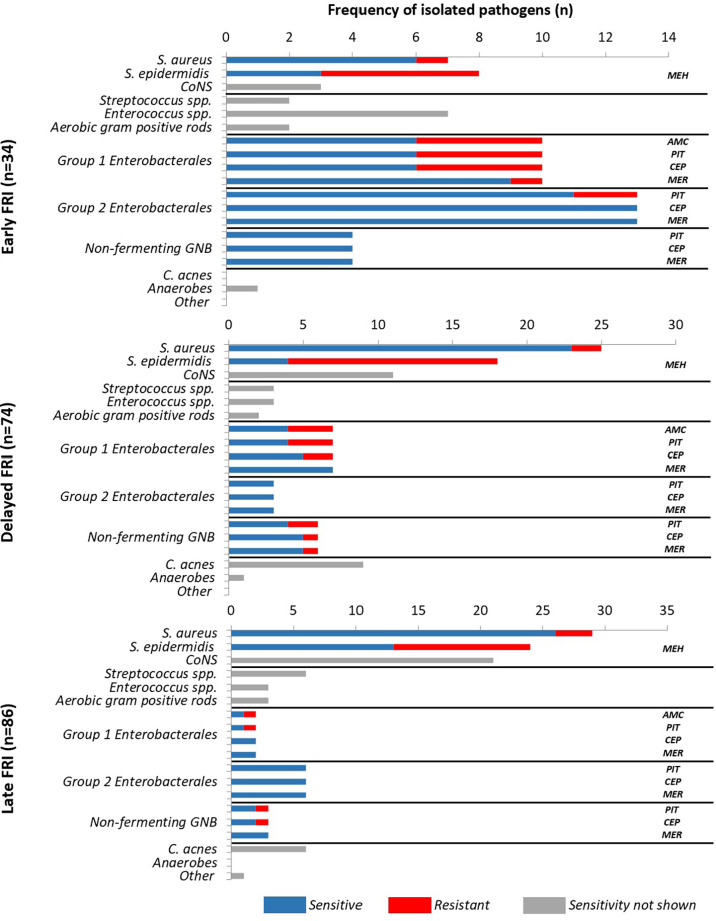
Antimicrobial susceptibility of pathogens in early, delayed and late fracture-related infections. (AMC, Amoxicillin/clavulanic acid; CEP, Cefepime; MEH, Methicillin; MER, Meropenem; PIT, Piperacillin-tazobactam).

**Figure 4** shows the microbiological epidemiology according to the interval from primary fracture fixation to onset of FRI and susceptibility for GNB and staphylococci to different β-lactam antibiotics. Resistance data was available for all isolated pathogens. Regardless of time to onset, the rate of methicillin-resistant *Staphylococcus* spp. was high (e.g. 60% methicillin-resistant *S. epidermidis)*, but susceptibility to vancomycin remained 100%. These results also show that 75.9% of GNB were sensitive to piperacillin/tazobactam. Susceptibility to cefepime and meropenem was higher, namely 85.2% and 96.3%, respectively.

## Discussion

Publications focusing on the microbiological epidemiology of FRI are still scarce. This is especially true for data on the relation between the microbiology and clinical signs or time to onset of infection. Therefore, the primary aim of our study was to evaluate the microbiological epidemiology of FRIs, including the association with clinical symptoms and antimicrobial susceptibility data. The secondary aim was to analyze whether there was a relationship between the Willenegger and Roth classification and the microbiological etiology of FRI to guide empirical antibiotic therapy. In brief, the present study revealed that in early FRI, polymicrobial, enterococcal and *Enterobacterales* etiologies were more frequent, indicating more frequent soil contamination in this type of FRI.

### Microbiological Epidemiology

Previous studies focusing on FRI reported a polymicrobial infection rate of approximately 30% in their patient cohorts, which is similar to our results (25.3%), but higher compared to PJI (10%) (Flurin et al., 2019; Kuehl et al., 2019; Wang et al., 2021). Furthermore, the predominant pathogens that are identified in the literature in case of polymicrobial infections are GNB (Kuehl et al., 2019; Rupp et al., 2020; Wang et al., 2021). In our study, GNB also predominated (65.3%), followed by non-*epidermidis* CoNS (44.9%), *S. epidermidis* (34.7%) and *S. aureus* (20.4%). Overall, GNB were isolated in 27.8% of the cases which is comparable to the 26.2% found by Kuehl *et al.* and higher than the 14% generally reported in PJI studies (Kuehl et al., 2019; Triffault-Fillit et al., 2019). *C. acnes* is the predominant microorganism in sebaceous follicles of the skin which is more frequently observed in the shoulder region than on the skin of the knee and hip (Hudek et al., 2021). This explains why in our cohort this pathogen was mainly isolated in FRIs of the upper extremity.

In our patient cohort with a monomicrobial FRI, the presence of a virulent pathogen was associated with the presence of pus or purulent drainage and the presence of a fistula.

### Antibiotic Susceptibility Data

At initial clinical presentation, a high bacterial load is commonly found at the infection site. Therefore, the risk for emergence of resistance is significant during this period, especially when fluoroquinolones or rifampicin are used (Zimmerli et al., 1998; Sendi and Zimmerli, 2012). In contrast, emergence of resistance against β-lactam antibiotics and vancomycin does not occur during treatment, even if the bacterial load is high (Sendi and Zimmerli, 2012). From this point of view, these antibiotic agents are qualified for use in an empirical setting. Currently, guidelines recommend the use of a glycopeptide in combination with a β-lactam antibiotic (Hoiby et al., 2015; Depypere et al., 2019a).

In our study, vancomycin susceptibility of staphylococci was 100%. Susceptibility of any type of microorganism to piperacillin/tazobactam was lower (75.9%) as compared to cefepime (85.2%) and meropenem (96.3%), respectively. Nevertheless, Piperacillin/tazobactam combined with vancomycin seems a rational initial option as mentioned in recent guidelines (Depypere et al., 2019a). Our study shows that cefepime could be a potential alternative as combination partner to vancomycin. Breilh *et al.* reported an excellent diffusion of cefepime into bone tissue, with concentrations in cancellous and cortical bone greater than the minimum concentrations required to inhibit growth of 90% of the strains (MIC_90_) (Breilh et al., 2003; Thabit et al., 2019). However, a diminished efficacy of cefepime for the treatment of extended-spectrum beta-lactamase (ESBL) infections with a high bacterial inoculum (i.e. osteomyelitis) has been shown in animal models (Karaiskos and Giamarellou, 2020). The ESCMID study group on multidrug resistant organisms recommends against the use of cefepime in case there is resistance to third generation cephalosporins (Paul et al., 2021). In our cohort, two pathogens would therefore no longer be eligible for cefepime therapy. This brings the percentage of cefepime coverage to 81.5% instead of 85.2%. A major advantage of cefepime is the proven stability against AmpC beta-lactamases (Siedner et al., 2014; Harris et al., 2016; Rodriguez-Bano et al., 2018; Tamma et al., 2021). A disadvantage is its lack of coverage against Gram negative anaerobes which surprisingly, were not found in our study population. This could be due to our current culturing methods which might not be sufficient for growth of anaerobic micro-organisms. Kuehl et al. (Kuehl et al., 2019) reported 16.3% anaerobes but did not differentiate between Gram positive and –negative cases. Two other studies also did not report the presence of anaerobes (Peng et al., 2017; Wang et al., 2021). Thus, it remains unclear whether anaerobic activity is required in case of empirical therapy. Considering only susceptibility data, meropenem would be the best option for empirical treatment in early, delayed and late FRI. However, misuse and overuse of carbapenems has resulted in the emergence of carbapenem-resistance which represents a paramount therapeutic challenge.

In addition, there is another critical concern. The current guidelines regarding empirical antibiotic therapy are not based on scientific data (Depypere et al., 2019a). Studies evaluating the need for empirical therapy in FRI are scarce (Hellebrekers et al., 2020). When patients have a severe life-threatening infection (e.g. sepsis) rapid and correct empirical therapy is proven to be crucial (Strich et al., 2020). Although FRI can lead to severe complications, it is not a life-threatening disease when the patient is not septic. Several critical questions arise regarding the use of empirical therapy. First, what are the consequences if empirical therapy is not started, but delayed targeted antibiotic therapy is initiated based on culture results? Second, is rapid empirical therapy needed for all patients with FRI at time of definitive fracture fixation? Third, does the need for empirical therapy depend on the type of surgical strategy (e.g. DAIR, one vs two- stage exchange, internal vs external fixation)? These questions are crucial and should be answered in large prospective multicenter studies.

### Microbiological Etiology According to Willenegger and Roth Classification

Traditionally, duration of infection is considered as one of the most important factors in the treatment decision making process of FRI. One of the reasons is that there is a decreasing antibiotic susceptibility with maturation of bacterial biofilms on implants over time (Costerton et al., 1999; Barberan et al., 2006; Metsemakers et al., 2018; Stewart and Costerton, 2001). Therefore, time after fracture fixation is the most commonly used classification for FRI (Willenegger and Roth, 1986; Metsemakers et al., 2018; Metsemakers et al., 2019). Various time-based classifications have directed surgeons towards one of the two main surgical principles in FRI treatment: debridement, antimicrobial therapy, and implant retention (DAIR) or debridement antimicrobial therapy and implant removal/exchange. One of the most used classifications was described by Willenegger and Roth who divided FRI into early, delayed, and late-onset infection with respective cut-offs after two and ten weeks (Willenegger and Roth, 1986). However, the evidence in the literature for a clear time-based cut-off to aid in the decision-making process between implant retention and removal is scarce. Morgenstern *et al.* recently published a systematic review in which they concluded that acute/early FRI successfully could be treated with DAIR up to 10 weeks after osteosynthesis (Morgenstern et al., 2021). As a result, the distinction between early and delayed is not meaningful in this setting. Other factors must be taken into account (e.g. construct stability, causative pathogen) for treatment success. Therefore, we investigated whether microbiological epidemiology depends on this time-based classification.

It was previously stated that early infections after osteosynthesis are mainly caused by virulent pathogens (e.g. *S. aureus*, β-haemolytic streptococci*, S. lugdunensis*, GNB) (McBride, 2010; Metsemakers et al., 2019). Our data showed that early infections were mostly caused by GNB (50.0%). *S. aureus* and *S. lugdunensis* were isolated in only 20.6% of the early FRIs, and in 39.5% of the late-onset FRIs. Unfortunately, documentation of haematogenous seeding is lacking, as is often the case in fracture-related infection studies. A few studies reported a predominance of *S. aureus* in each time interval (Kuehl et al., 2019; Baertl et al., 2022), whereas in our study, *S. aureus* predominated in the delayed and late onset group.

A recent paper evaluated empirical antibiotic therapy according to onset of FRI. No significant differences in the potential efficacy of empiric antimicrobial regimens were observed between early, delayed and late-onset FRI, except for early FRI, in which the combination ciprofloxacin and glycopeptide was superior as compared to delayed and late FRI (Baertl et al., 2022). Fluoroquinolone susceptibility was not evaluated in our cohort, because selection of resistance to these agents is possible when the bioburden is high, which makes them not suitable as empirical agent (Greenberg et al., 1987; Aboltins et al., 2011).

### Limitations

Several limitations regarding our study should be mentioned. First, the study design was retrospective, leading to a reduced level of evidence and difficult interpretation. Second, we performed a single-center study. Thus, the microbiological spectrum and the susceptibility pattern reflects a local situation. A large multicenter study would offer more information and would increase the study’s scientific value. However, different diagnostic culture protocols between centers would make interpretation of the results difficult. A third limitation is the knowledge gap regarding the use of empirical therapy in FRI.

## Conclusions

This study revealed that in early FRIs, polymicrobial infections and infections including Enterobacterales and enterococcal species were more frequent. A time-based FRI classification is not meaningful to estimate the microbiological epidemiology and cannot be used to guide empiric antibiotic therapy. Large multicenter prospective studies are necessary to gain more insight into the added value of (broad) empirical antibiotic therapy.

## Data Availability Statement

The raw data supporting the conclusions of this article will be made available by the authors, without undue reservation.

## Ethics Statement

The studies involving human participants were reviewed and approved by the Ethics Committee of the University Hospitals Leuven, Belgium (S62394). Written informed consent for participation was not required for this study in accordance with the national legislation and the institutional requirements.

## Author Contributions

MD, JS, JO, WM contributed to the study conception and design. Material preparation, data collection and analysis were performed by JS, MD and JO. The first draft of the manuscript was written by MD, JS and WM. JO, YD, GG, FIJ and WZ commented on subsequent versions of the manuscript. All authors read and approved the final manuscript.

## Funding

This research received no specific grant from any funding agency in the public, commercial, or not-for-profit sectors.

## Conflict of Interest

The authors declare that the research was conducted in the absence of any commercial or financial relationships that could be construed as a potential conflict of interest.

## Publisher’s Note

All claims expressed in this article are solely those of the authors and do not necessarily represent those of their affiliated organizations, or those of the publisher, the editors and the reviewers. Any product that may be evaluated in this article, or claim that may be made by its manufacturer, is not guaranteed or endorsed by the publisher.
